# Association of Fundus Autofluorescence Abnormalities and Pachydrusen in Central Serous Chorioretinopathy and Polypoidal Choroidal Vasculopathy

**DOI:** 10.3390/jcm11185340

**Published:** 2022-09-11

**Authors:** Timothy Y. Y. Lai, Ziqi Tang, Adrian C. W. Lai, Simon K. H. Szeto, Ricky Y. K. Lai, Carol Y. Cheung

**Affiliations:** 1 Department of Ophthalmology & Visual Sciences, The Chinese University of Hong Kong, Hong Kong, China; 2 2010 Retina & Macula Centre, Tsim Sha Tsui, Kowloon, Hong Kong, China; 3 Faculty of Medicine & Health, UNSW Sydney, Kensington, NSW 2052, Australia

**Keywords:** pachychoroid, pachydrusen, drusen, central serous chorioretinopathy, polypoidal choroidal vasculopathy, optical coherence tomography, fundus autofluorescence, neovascular age-related macular degeneration

## Abstract

A specific form of drusen, known as pachydrusen, has been demonstrated to be associated with pachychoroid eye diseases, such as central serous chorioretinopathy (CSC) and polypoidal choroidal vasculopathy (PCV). These pachydrusen have been found in up to 50% of eyes with CSC and PCV and may affect the disease progression and treatment response. This study aims to investigate the association between pachydrusen and changes in fundus autofluorescence (FAF) in eyes with CSC and PCV. A total of 65 CSC patients and 32 PCV patients were evaluated. Pachydrusen were detected using both color fundus photography and spectral-domain optical coherence tomography. The relationships between pachydrusen and FAF changes were then investigated. The prevalence of pachydrusen in CSC and PCV eyes was 16.7% and 61.8%, respectively. The mean age of patients with pachydrusen was significantly older than those without pachydrusen (CSC: 56.3 vs. 45.0 years, *p* < 0.001; PCV: 68.8 vs. 59.5 years, *p* < 0.001). No significant difference was found in the mean subfoveal choroidal thickness between eyes with or without pachydrusen. Eyes with pachydrusen were significantly associated with more extensive FAF changes in both CSC and PCV (*p* < 0.001 and *p* = 0.037, respectively). The study demonstrated that pachydrusen are more prevalent in PCV than CSC. Increasing age and more extensive abnormalities in FAF are associated with the presence of pachydrusen, suggesting that dysfunction of retinal pigment epithelial cells is associated with pachydrusen.

## 1. Introduction

Pachychoroid eye diseases are eye conditions that are associated with localized thickening of choroid with dilated or congested choroidal veins, reduced or absent choriocapillaris, causing progressive dysfunction of the retinal pigment epithelia (RPE), and may lead to macular neovascularization [1,2]. Conditions classified in this spectrum of pachychoroid eye diseases include pachychoroid pigment epitheliopathy, central serous chorioretinopathy (CSC), polypoidal choroidal vasculopathy (PCV), focal choroidal excavation, pachychoroid neovasculopathy, and peripapillary pachychoroid syndrome [1,2].

One of the clinical features more commonly detected in patients with pachychoroid eye diseases is a specific type of drusen known as pachydrusen [3,4]. These pachydrusen are larger than 125 µm, more asymmetrical in shape with irregular outer contour, have a diffuse and more widespread distribution over the posterior pole that spares the central macula, and can occur in multiple groups of several deposits or in isolation [3]. Pachydrusen can be diagnosed using multimodal imaging including color fundus photographs, spectral-domain optical coherence tomography (OCT) and enhanced depth imaging OCT [3]. The prevalence of pachydrusen in patients with pachychoroid eye diseases appear to vary among different eye conditions and ethnic groups, with reported rates of 6.8% to 60% in eyes with CSC [5,6,7,8], and 14.1% to 56% in eyes with PCV [8,9,10,11]. With the use of multimodal imaging including indocyanine green angiography (ICGA) and OCT B-scans and en face images, it has been demonstrated that the majority of pachydrusen are located over pachyvessels (dilated Haller vessels) and concentrated within areas of geographic filling delay of choriocapillaris [5,12]. These pachydrusen are known to be of specific prognostic significance, as they have been demonstrated to be associated with progression to PCV but not to typical neovascular age-related macular degeneration (AMD) [13,14].

Fundus autofluorescence (FAF) is a non-invasive imaging technique that makes use of light stimulation of lipofuscin in the RPE, and changes in the levels of FAF emitted have the potential to assess the RPE functional activity [15]. Several studies have evaluated the changes in FAF in eyes with pachychoroid diseases and have demonstrated various severity of FAF abnormalities due to RPE dysfunction [16,17,18,19,20,21,22]. However, the association between pachydrusen and functional imaging using FAF has not been evaluated previously. We postulated that pachydrusen might be associated with the dysfunction of RPE demonstrated by FAF. The aim of this study is to evaluate the prevalence of pachydrusen and to assess the association between FAF changes and pachydrusen in eyes with CSC and PCV.

## 2. Materials and Methods

### 2.1. Study Design

This was a retrospective study of consecutive patients with newly diagnosed treatment-naïve CSC or PCV referred for fluorescein angiography (FA) and ICGA performed in the 2010 Retina and Macula Centre, Hong Kong from January 2013 to December 2018. The inclusion criteria of the study included: CSC or PCV diagnosed based on existing definitions [1,2]; and no prior treatment for macular or retinal diseases including anti-vascular endothelial growth factor (anti-VEGF) injection, verteporfin photodynamic therapy and thermal laser photocoagulation. Exclusion criteria included: high myopia (spherical equivalent refractive error < −6 diopters); other co-existing retinal or macular diseases such as epiretinal membrane, macular hole, diabetic retinopathy, retinal vascular occlusion, posterior uveitis, retinal detachment; and media opacity affecting ophthalmic imaging quality. The study was conducted in accordance with the Declaration of Helsinki.

### 2.2. Imaging and Image Analyses

All patients underwent fundus photography, FAF, FA, and ICGA using the flash-based TRC-50DX retinal camera (Topcon, Tokyo, Japan). Spectral-domain OCT scans were obtained using with the Cirrus HD-OCT 4000 (Carl Zeiss Meditec, Dublin, CA, USA) with the enhanced depth imaging (EDI) mode. Subfoveal choroidal thickness (SFCT) was measured using the horizontal and vertical OCT B-scan EDI images at the fovea with the caliper measurement tool within the OCT system software. SFCT was measured from the outer surface of the RPE band to the inner surface of the choroidal-scleral interface at the fovea [11]. Pachydrusen were diagnosed based on the following criteria: drusen diameter > 125 µm; irregular outer contours of the drusen; and drusen occurring in isolation or in groups [3,5]. The area of pachydrusen (mm^2^) was measured, as in the fundus photographs, using an imaging software (ImageJ version 1.53a, National Institutes of Health, Bethesda, MD, USA) [23]. The area of FAF abnormality was measured based on the sum of all sites of FAF abnormalities in the FAF images and was classified as <2 disc areas or ≥2 disc areas.

### 2.3. Statistical Analyses

All data were entered into a computer spreadsheet software (Mircosoft Excel for Mac version 16.54, Mircosoft Corp, Redmond, WA, USA) and statistical analyses were carried out using a statistical module (StatPlus:mac Pro Core version 5.9.80, AnalystSoft Inc., Walnut, CA, USA) running within the spreadsheet software. Descriptive data were summarized as mean ± standard deviation (SD) or percentages. Comparisons between eyes with or without pachydrusen were performed using two-tailed *t*-test (for continuous variables) or chi-squared test (for categorical variables). A *p*-value of ≤0.05 was considered as statistically significant.

## 3. Results

### 3.1. Patient Demongraphics

A total of 72 eyes of 65 CSC patients and 34 eyes of 32 PCV patients was included. Seven patients had bilateral CSC and two patients had bilateral PCV. For the 65 CSC patients, the mean ± SD age was 46.7 ± 8.8 years (range, 29 to 69 years) and there were 50 (76.9%) males and 15 (23.1%) females. For the 32 PCV patients, the mean ± SD age was 65.0 ± 8.2 years (range, 44 to 82 years) and there were 23 (71.9%) males and 9 (28.1%) females.

### 3.2. Eyes with Central Serous Chorioretinopathy

**Figure 1:**
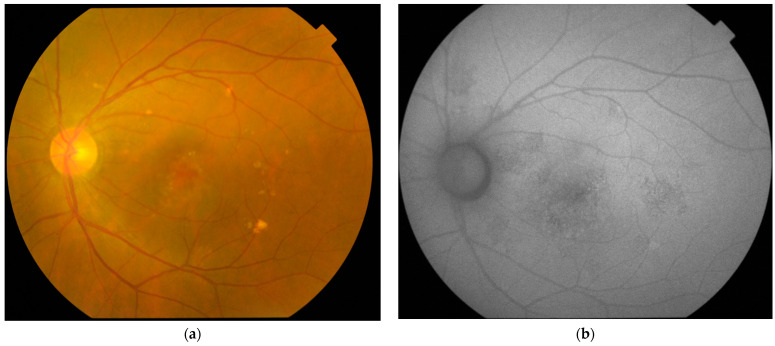
(**a**) Fundus photograph of a 69-year-old patient with central serous chorioretinopathy showing multiple pachydrusen in the inferiotemporal and temporal macula occurring as a cluster and scattered in the superior macula around the vascular arcade; (**b**) fundus autofluorescence showing mixed increased and reduced autofluorescence of ≥2 disc areas scattered in the macula due to widespread dysfunction of the retinal pigment epithelium.

Pachydrusen were found in 12 (16.7%) of the 72 eyes with CSC and the mean ± SD area of pachydrusen was 0.20 ± 0.26 mm^2^. The mean ± SD age of 10 CSC patients with pachydrusen was significantly older than the 55 CSC patients without pachydrusen, with 56.3 ± 7.8 years and 45.0 ± 7.8 years, respectively (two-tailed *t*-test, *p* < 0.001) (Table 1). For the seven patients with bilateral CSC, five (71.4%) had no pachydrusen in both eyes, one (14.3%) had pachydrusen in one eye, and one (14.3%) had pachydrusen in both eyes. The mean ± SD SFCT for all CSC eyes was 359.2 ± 42.9 µm. There was no significant difference between the mean ± SD SFCT of CSC eyes with or without pachydrusen (two-tailed *t*-test, *p* = 0.38). FAF abnormalities were found in 71 (98.6%) of 72 eyes with CSC, with 37 (51.4%) eyes having < 2 disc areas of FAF abnormality, and 34 (47.2%) eyes having ≥ 2 disc areas of FAF abnormality. Eyes with pachydrusen were found to have significantly more extensive area of FAF abnormality with ≥ 2 disc areas than eyes without pachydrusen (chi-squared test, *p* < 0.001). An example of a CSC eye with pachydrusen and FAF abnormalities is displayed in Figure 1.

### 3.3. Eyes with Polypoidal Choroidal Vasculopathy

**Figure 2:**
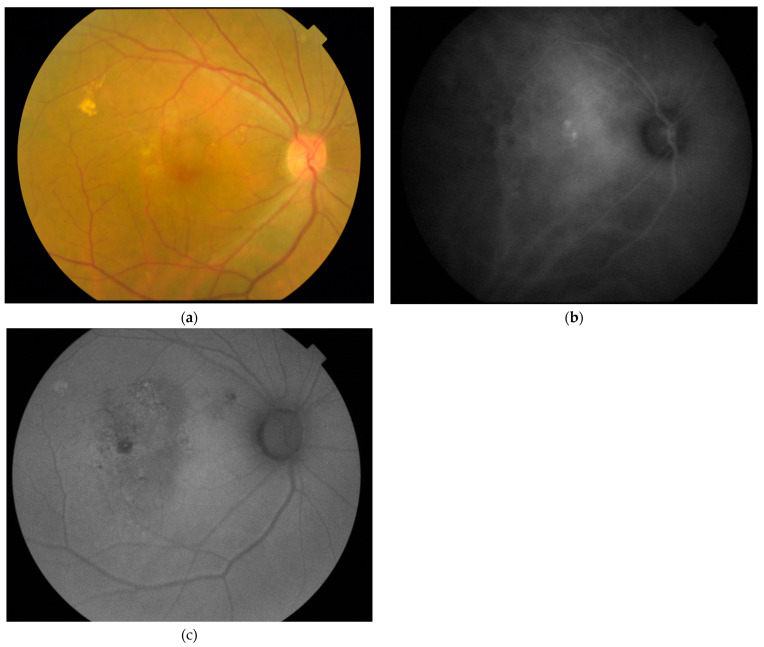
(**a**) Fundus photo of a 65-year-old patients with polypoidal choroidal vasculopathy showing multiple pachydrusen occurring as a cluster in the superiotemporal macula with a few small isolated drusen in the superionasal retina; (**b**) indocyanine green angiography showing multiple polypoidal lesions at the macula with dilated choroidal vasculature and choroidal hyperpermeability; (**c**) fundus autofluorescence showing mixed autofluorescence abnormalities with increase and reduced autofluorescence of ≥2 disc areas scattered in the central macula with a ring of increased autofluorescence highlighting the polypoidal lesions.

Pachydrusen was found in 21 (61.8%) of the 34 eyes with PCV and the mean ± SD area of pachydrusen was 0.32 ± 0.60 mm^2^. Similar to CSC, the mean age of the 19 PCV patients with pachydrusen was significantly older than the 13 PCV patients without pachydrusen, with 68.8 years and 59.5 years, respectively (two-tailed *t*-test, *p* < 0.001) (Table 2). For the two patients with bilateral PCV, both patients had pachydrusen in both eyes. The mean ± SD SFCT for all PCV eyes was 264.5 ± 68.0 µm. No significant difference between the mean ± SD SFCT of PCV eyes with or without pachydrusen was observed (two-tailed *t*-test, *p* = 0.88). FAF abnormalities were found in all 33 (100%) eyes with PCV, with 5 (14.7%) eyes having < 2 disc areas of FAF abnormality and 29 (85.3%) eyes having ≥ 2 disc areas of FAF abnormality. The proportion of eyes with FAF abnormality of ≥ 2 disc areas was significantly higher in eyes with PCV than CSC (chi-squared test, *p* = 0.001). There was also a significant association between the presence of pachydrusen and ≥ 2 disc areas extent of FAF abnormality (chi-squared test, *p* = 0.037). An example of a PCV eye with pachydrusen and FAF abnormalities is displayed in Figure 2.

## 4. Discussion

In this study, we evaluated the prevalence of pachydrusen in eyes with CSC and PCV and the prevalence was found to be 16.7% and 61.8%, respectively. A previous study has demonstrated an ethnic link in the prevalence of pachydrusen, as pachydrusen were significantly more common in Asian neovascular AMD patients compared with white patients [24]. Our rate of pachydrusen in CSC eyes appeared to be slightly lower compared with most previous studies performed in Asian populations [5,6,7,8]. The reported prevalence of pachydrusen in CSC eyes were 27.2% and 40.1% in two studies conducted in Japan [5,7], and 6.8% and 60% in two studies performed in India [6,8]. However, our rate appeared similar to the pachydrusen prevalence of 20% reported by Kim et al. in Korea [25]. The main reason for the lower prevalence of pachydrusen in our CSC patients is likely due to the younger mean age of patients in our study compared with other studies. In our study, the mean age of CSC patients was 46.7 years, whereas studies with higher prevalence of pachydrusen than our study all had a mean age of >50 years [5,7,8]. A study that reported a lower prevalence of pachydrusen in CSC eyes than our study had a mean age of 42.9 years [6], which was younger than our study. The influence of age on the presence of pachydrusen can be demonstrated in our study as well as in previous studies [5,7,8,25], since the mean age of CSC patients with pachydrusen was significantly higher than those without pachydrusen. Takahasi et al. reported the prevalence of pachydrusen in CSC patients was 40.1% and CSC patients with pachydrusen were significantly older than those without, with a mean age of 62.1 years versus 48.8 years, respectively [7]. For PCV, the influence of age on the prevalence of pachydrusen could also be observed, as we also found that patients with pachydrusen were significantly older than those without pachydrusen. Therefore, increasing age appeared to be an important factor for the development of pachydrusen in both CSC and PCV.

FAF is an investigation that can provide in vivo evaluation of RPE function, and abnormalities in FAF are common findings in both CSC and PCV eyes [26,27,28]. In our study, FAF abnormalities were found in 98.6% of eyes with CSC and in all eyes with PCV. The proportion of eyes with FAF abnormality of ≥ 2 disc areas was higher in eyes with PCV than CSC, with 85.3% vs. 47.2%, respectively. We also found that both PCV and CSC eyes with pachydrusen were associated with more extensive RPE dysfunction as demonstrated by FAF abnormalities. The findings suggest pachydrusen might be an indicator of more severe RPE dysfunction and eyes with PCV are associated with more extensive RPE dysfunction compared with CSC. Eyes with PCV generally have larger area of RPE and photoreceptor damage due to macular hemorrhage, exudation, and multiple recurrent pigment epithelial detachments. Therefore, it is likely that PCV eyes will have more extensive RPE dysfunction, resulting in larger area of FAF abnormalities.

There are several limitations associated with our study, including the relatively small sample size and the lack of longitudinal follow-up of the patients. Due to the small sample size, we did not perform multivariate analysis such as regression analysis to evaluate the potential confounding effects on other variables, such as SFCT. In our study, the mean SFCT was found to be similar in eyes with or without pachydrusen in both CSC and PCV eyes. In a large prospective study of over 600 eyes with treatment-naive CSC by Takahasi et al. [7], although the mean SFCT was similar between eyes with or without pachydrusen, SFCT was found to be significantly thicker in eyes with pachydrusen than those without pachydrusen after adjusting for age, gender, and refractive error in multivariate analysis. Notomi et al. also demonstrated that in eyes with pachydrusen and intermediate size drusen, there was a trend of thicker SFCT in these eyes compared with eyes without pachydrusen [29]. However, in the study by Kim et al. [25], pachydrusen was only associated with choroidal thickness in eyes with PCV and not in CSC. Therefore, the interaction between the presence of pachydrusen and choroidal thickness warrants further investigations. In addition, we did not formally evaluate the precise relationship between the location of the pachydrusen and FAF abnormalities. Nonetheless, since the area of FAF abnormality was considerably larger and more widespread than that of pachydrusen, there appeared to be a lack of relationship between the location of the pachydrusen and FAF abnormalities.

Previous follow-up studies have demonstrated that pachydrusen might have important prognostic implications for the progression of PCV [13,14]. In a longitudinal study by Teo et al. [13], the authors evaluated the natural course of 29 eyes with pachydrusen and found that eyes with pachydrusen were significantly more likely to develop into PCV rather than typical neovascular AMD. Similar findings were also observed in a retrospective cohort study by Kim et al. [14], in which 11.5% of 61 eyes with pachydrusen developed into PCV after 5 years, while only 3.3% developed into typical neovascular AMD. In addition to the association with natural history, pachydrusen have also been demonstrated to be a predictor in the treatment response in PCV patients receiving intravitreal anti-VEGF monotherapy [28]. In a retrospective cohort study by Fukuda et al. [30], patients with pachydrusen in the fellow eye had significantly fewer additional intravitreal aflibercept injections following the initial three loading doses for the treatment of PCV. However, similar studies on the influence of pachydrusen on the natural history or treatment outcome have not been performed in CSC. Further longitudinal studies to evaluate the prognostic implications of pachydrusen, especially in eyes with CSC, are therefore warranted. Future studies can also explore the possible association between pachydrusen and persistent subretinal fluid in CSC and PCV eyes following treatment.

## 5. Conclusions

Pachydrusen are associated with increasing age and have higher prevalence in eyes with PCV than CSC. A more extensive area of FAF abnormality was found in eyes with pachydrusen, suggestive of more widespread RPE dysfunction in eyes with the presence of pachydrusen.

## Figures and Tables

**Table 1:** Characteristics of CSC eyes with or without pachydrusen.

Characteristics	All CSC Eyes (*n* = 72)	With Pachydrusen (*n* = 12)	Without Pachydrusen (*n* = 60)	*p* Value
Mean ± SD age (years)	46.7 ± 8.8	56.3 ± 7.8	45.0 ± 7.8	<0.001 ^1^
Mean ± SD SFCT (µm)	359.2 ± 42.9	348.5 ± 33.8	361.8 ± 44.8	0.38 ^2^
FAF abnormality None <2 disc areas ≥2 disc areas	1 (1.4%) 37 (51.4%) 34 (47.2%)	0 (0.0%) 0 (0.0%) 12 (100.0%)	1 (1.7%) 37 (61.7%) 32 (36.7%)	<0.001 ^3^

SD: standard deviation; CSC: central serous chorioretinopathy; SFCT: subfoveal choroidal thickness; FAF: fundus autofluorescence. ^1^ two-tailed *t*-test between patients with pachydrusen vs. without pachydrusen. ^2^ two-tailed *t*-test between eyes with pachydrusen vs. without pachydrusen. ^3^ chi-square test between eyes with pachydrusen vs. without pachydrusen.

**Table 2:** Characteristics of PCV eyes with or without pachydrusen.

Characteristics	All PCV eyes (*n* = 34)	With Pachydrusen (*n* = 21)	Without Pachydrusen (*n* = 13)	*p* Value
Mean ± SD age (years)	65.0 ± 8.2	68.8 ± 7.2	59.5 ± 6.6	< 0.001 ^1^
Mean ± SD SFCT (µm)	264.5 ± 68.0	262.8 ± 70.0	267.0 ± 68.7	0.88 ^2^
FAF abnormality <2 disc areas ≥2 disc areas	5 (14.7%) 29 (85.3%)	1 (4.8%) 20 (95.2%)	4 (30.8%) 9 (69.2%)	0.37 ^3^

SD: standard deviation; CSC: central serous chorioretinopathy; SFCT: subfoveal choroidal thickness; FAF: fundus autofluorescence. ^1^ two-tailed *t*-test between patients with pachydrusen vs. without pachydrusen. ^2^ two-tailed *t*-test between eyes with pachydrusen vs. without pachydrusen. ^3^ chi-square test between eyes with pachydrusen vs. without pachydrusen.

## Data Availability

The data presented in this study are available on request from the corresponding author. The data are not publicly available due to privacy.

## References

[1] Cheung C.M.G., Lee W.K., Koizumi H., Dansingani K., Lai T.Y.Y., Freund K.B. (2019). Pachychoroid disease. Eye.

[2] Castro-Navarro V., Behar-Cohen F., Chang W., Joussen A.M., Lai T.Y.Y., Navarro R., Pearce I., Yanagi Y., Okada A.A. (2021). Pachychoroid: Current concepts on clinical features and pathogenesis. Graefes Arch. Clin. Exp. Ophthalmol..

[3] Spaide R.F. (2018). Disease expression in nonexudative age-related macular degeneration varies with choroidal thickness. Retina.

[4] Zhang X., Sivaprasad S. (2021). Drusen and pachydrusen: The definition, pathogenesis, and clinical significance. Eye.

[5] Matsumoto H., Mukai R., Morimoto M., Tokui S., Kishi S., Akiyama H. (2019). Clinical characteristics of pachydrusen in central serous chorioretinopathy. Graefes Arch. Clin. Exp. Ophthamlol..

[6] Singh S.R., Chakurkar R., Goud A., Chhablani J. (2020). Low incidence of pachydrusen in central serous chorioretinopathy in an Indian cohort. Indian J. Ophthalmol..

[7] Takahashi A., Hosoda Y., Miyake M., Miyata M., Oishi A., Tamura H., Ooto S., Yamashiro K., Tabara Y., Matsuda F. (2021). Clinical and genetic characteristics of pachydrusen in eyes with central serous chorioretinopathy and general Japanese individuals. Ophthalmol. Retin..

[8] Sheth J., Anantharaman G., Kumar N., Parachuri N., Bandello F., Kuppermann B.D., Loewenstein A., Sharma A. (2020). Pachydrusen: The epidemiology of pachydrusen and its relevance to progression of pachychoroid disease spectrum. Eye.

[9] Singh S.R., Chakurkar R., Goud A., Rasheed M.A., Vupparaboina K.K., Chhablani J. (2019). Pachydrusen in polypoidal choroidal vasculopathy in an Indian cohort. Indian J. Ophthalmol..

[10] Lee J., Byeon S.H. (2019). Prevalence and clinical characteristics of pachydrusen in polypoidal choroidal vasculopathy: Multimodal image study. Retina.

[11] Lee J., Kim M., Lee C.S., Kim S.S., Koh H.J., Lee S.C., Byeon S.H. (2020). Drusen subtypes and choroidal characteristics in Asian eyes with typical neovascular age-related macular degeneration. Retina.

[12] Baek J., Lee J.H., Chung B.J., Lee K., Lee W.K. (2019). Choroidal morphology under pachydrusen. Clin. Exp. Ophthalmol..

[13] Teo K., Cheong K.X., Ong R., Hamzah H., Yanagi Y., Wong T.Y., Chakravarthy U., Cheung C. (2021). Macular neovascularization in eyes with pachydrusen. Sci. Rep..

[14] Kim K.L., Joo K., Park S.J., Park K.H., Woo S.J. (2022). Progression from intermediate to neovascular age-related macular degeneration according to drusen subtypes: Bundang AMD cohort study report 3. Acta Ophthalmol..

[15] Schmitz-Valckenberg S., Pfau M., Fleckenstein M., Staurenghi G., Sparrow J.R., Bindewald-Wittich A., Spaide R.F., Wolf S., Sadda S.R., Holz F.G. (2021). Fundus autofluorescence imaging. Prog. Retin. Eye Res..

[16] Margolis R., Mukkamala S.K., Jampol L.M., Spaide R.F., Ober M.D., Sorenson J.A., Gentile R.C., Miller J.A., Sherman J., Freund K.B. (2011). The expanded spectrum of focal choroidal excavation. Arch. Ophthalmol..

[17] Warrow D.J., Hoang Q.V., Freund K.B. (2013). Pachychoroid pigment epitheliopathy. Retina.

[18] Pang C.E., Freund K.B. (2015). Pachychoroid neovasculopathy. Retina.

[19] Zhao X., Xia S., Chen Y. (2018). Characteristic appearances of fundus autofluorescence in treatment-naive and active polypoidal choroidal vasculopathy: A retrospective study of 170 patients. Graefes Arch. Clin. Exp. Ophthalmol..

[20] van Rijssen T.J., van Dijk E.H.C., Yzer S., Ohno-Matsui K., Keunen J.E.E., Schlingemann R.O., Sivaprasad S., Querques G., Downes S.M., Fauser S. (2019). Central serous chorioretinopathy: Towards an evidence-based treatment guideline. Prog. Retin. Eye Res..

[21] Han J., Cho N.S., Kim K., Kim E.S., Kim D.G., Kim J.M., Yu S.Y. (2020). Fundus autofluorescence patterns in central serous chorioretinopathy. Retina.

[22] Kumar V., Azad S.V., Verma S., Surve A., Vohra R., Venkatesh P. (2022). Peripapillary pachychoroid syndrome: New insights. Retina.

[23] Schneider C.A., Rasband W.S., Eliceiri K.W. (2012). NIH Image to ImageJ: 25 years of image analysis. Nat. Methods.

[24] Cheung C., Gan A., Yanagi Y., Wong T.Y., Spaide R. (2018). Association between choroidal thickness and drusen subtypes in age-related macular degeneration. Ophthalmol. Retin..

[25] Kim Y.H., Chung Y.R., Kim C., Lee K., Lee W.K. (2022). The association of pachydrusen characteristics with choroidal thickness and patient’s age in polypoidal choroidal vasculopathy versus central serous chorioretinopathy. Int. J. Mol. Sci..

[26] Govindahari V., Singh S.R., Rajesh B., Gallego-Pinazo R., Marco R.D., Nair D.V., Nair U., Chhablani J. (2019). Multicolor imaging in central serous chorioretinopathy—A quantitative and qualitative comparison with fundus autofluorescence. Sci. Rep..

[27] Shinojima A., Ozawa Y., Uchida A., Nagai N., Shinoda H., Kurihara T., Suzuki M., Minami S., Negishi K., Tsubota K. (2021). Assessment of hypofluorescent foci on late-phase indocyanine green angiography in central serous chorioretinopathy. J. Clin. Med..

[28] Yamagishi T., Koizumi H., Yamazaki T., Kinoshita S. (2012). Fundus autofluorescence in polypoidal choroidal vasculopathy. Ophthalmology.

[29] Notomi S., Shiose S., Ishikawa K., Fukuda Y., Kano K., Mori K., Wada I., Kaizu Y., Matsumoto H., Akiyama M. (2021). Drusen and pigment abnormality predict the development of neovascular age-related macular degeneration in Japanese patients. PLoS ONE.

[30] Fukuda Y., Sakurada Y., Sugiyama A., Yoneyama S., Matsubara M., Kikushima W., Tanabe N., Parikh R., Kashiwagi K. (2020). Pachydrusen in fellow eyes predict response to aflibercept monotherapy in patients with polypoidal choroidal vasculopathy. J. Clin. Med..

